# Systematic Review on the Impact of Salt-Reduction Initiatives by Socioeconomic Position to Address Health Inequalities in Adult Populations

**DOI:** 10.1093/nutrit/nuae088

**Published:** 2024-07-08

**Authors:** Ana Contreras Navarro, Kerrie Gallagher, Sally Griffin, Clarissa L Leydon, Ivan J Perry, Janas M Harrington

**Affiliations:** Centre for Health and Diet Research, School of Public Health, University College Cork, Cork, Ireland; Centre for Health and Diet Research, School of Public Health, University College Cork, Cork, Ireland; Centre for Health and Diet Research, School of Public Health, University College Cork, Cork, Ireland; Centre for Health and Diet Research, School of Public Health, University College Cork, Cork, Ireland; Centre for Health and Diet Research, School of Public Health, University College Cork, Cork, Ireland; Centre for Health and Diet Research, School of Public Health, University College Cork, Cork, Ireland

**Keywords:** nutrition policy, health equity, diet, sodium, social determinants of health

## Abstract

**Context:**

International evidence shows that individuals from low socioeconomic positions (SEPs) consume a greater amount of salt than those from higher SEPs. This health inequality reflects a disproportionate effect of salt-reduction initiatives, and explains a higher prevalence of cardiovascular disease among the most vulnerable populations. Assessing this impact can help tailor implementation strategies in the future for the benefit of the whole population.

**Objective:**

The aim was to systematically review the literature and assess the impact of salt-reduction initiatives on health and behavioral outcomes of adults by SEP.

**Data Sources:**

The search strategy was conducted in 6 databases (CINAHL, Scopus, Embase, MEDLINE, PubMed, and Web of Science) using the terms sodium or salt, social class, policy, intervention or campaign. Peer-reviewed articles assessing salt-reduction interventions in adults reporting dietary or behavioral changes on salt consumption measurements by SEP were considered for inclusion. Articles in which salt intake data were not reported by SEP were excluded.

**Data Extraction:**

Two reviewers collected data independently using a predesigned electronic form. The AXIS and RoB 2 tools were used for critical appraisal.

**Data Analysis:**

Eight studies containing data from 111 548 adults were interpreted according to study design following a narrative synthesis approach.

**Results:**

Salt-reduction initiatives are effective at reducing the intake of salt and sodium in adults. When reporting the impact of these initiatives, research outcomes are generally not evaluated by SEP, representing a question yet to be explored.

**Conclusion:**

A small number of articles that focused on the impact of salt-reduction interventions reported salt consumption measurements by SEP, indicating a critical gap in research. The limited evidence suggests potentially greater health benefits to be gained from the implementation of population-wide initiatives in adults of low SEP.

**Systematic Review Registration:**

PROSPERO registration no. CRD42021238055.

## INTRODUCTION

Excessive sodium consumption (>2 g/day) is a dietary and behavioral risk factor for elevated blood pressure and many noncommunicable diseases (NCDs), including stomach cancer, heart disease, and stroke.^1^^,^^2^ Over several decades, NCDs have remained among the leading causes of global mortality among adults, contributing to 41 million deaths every year. Cardiovascular diseases (CVDs) in particular, which involve a dose–response relationship between sodium consumption and blood pressure, account for 44% of deaths caused by NCDs.^3^^,^^4^ Recent estimates from the Global Burden of Disease study suggest that more than half of diet-related deaths and two-thirds of diet-related disability-adjusted life-years (DALYs) were attributable to a high intake of sodium.^5^

Globally, the dietary sources that contribute to a high intake of salt are bread and bakery products, cereals and grains, meat products, and dairy products.^6^ Sodium is found naturally in salt, with 2.5 g of salt containing 1.0 g of sodium. Recent estimates by the World Health Organization (WHO) suggest a global average sodium intake estimated at 4.310 g/day (10.78 g of salt/day). In its most recent global report on sodium intake reduction, the WHO recommended a set of immediate, population-based actions, including food reformulation to lower sodium content, implementing front-of-pack labeling to assist consumers in their food choices, conducting mass media campaigns to influence consumer knowledge and behavior, and implementing food-procurement policies to reduce sodium content in food service settings.^7^

### Background for salt reduction initiatives

In 2013, salt-reduction initiatives were prioritized at the 66th World Health Assembly. Thereafter, the Global Action Plan for the Prevention and Control of NCDs 2013–2020 included a list of 9 voluntary targets to address NCD risk factors, notably a “30% relative reduction in mean population intake of salt/sodium” and a “25% relative reduction in the prevalence of raised blood pressure or contain the prevalence of raised blood pressure.”^8^ To provide technical guidance and assist member states in the attainment of these targets, WHO developed the “SHAKE technical package for salt reduction” and proposed global benchmarks for sodium levels across food categories.^9^^,^^10^ In terms of regional CVD prevention strategy, WHO Europe launched an initiative for better heart health that involves a comprehensive policy approach. Launched on December 9, 2022, the CVD prevention initiative promotes food reformulation, setting national salt targets, restricting marketing of unhealthy foods to children, using front-of-pack labeling, and engaging in communication campaigns.^11^

The policy tools provided by WHO headquarters, WHO Europe, and other regional offices of WHO (eg, the Pan American Health Organization) are readily available to guide policymakers in the implementation of multicomponent prevention strategies involving not only statutory bodies for the development, enforcement, evaluation, and revision of salt-reduction initiatives but by integrating actions of key stakeholders influencing the characteristics and dynamics of the commercial food environment into national health-protecting policy action.^12^^,^^13^ Furthermore, in the global frame of the Sustainable Development Goals, the formulation and implementation of salt-reduction initiatives call for the simultaneous reduction in salt intake and the mitigation of existing health inequalities.^14^

### Inequities in salt and sodium intake

Inequities in the distribution of salt consumption indicate that individuals in a lower socioeconomic position (SEP) have a greater salt intake (5%–10% more) than those in a higher SEP.^1^ With regard to the health effects of excessive salt intake, recent data analysis reflects a disproportionate burden of CVD among low-income countries and among individuals with low SEP within Western countries.^15–17^ The term “socioeconomic position” refers to “the social and economic factors that explain the position that individuals or groups hold within the structure of society” and that may help us understand differences in health status and dietary risk factors observed between SEP groups.^18^ Measures of SEP can be drawn at the individual level (eg, occupation, education, and wealth) or at the neighborhood level (eg, poverty area indicators and housing characteristics).

Research focused on understanding the social determinants of health and diets has identified a complex network of factors embedded within societal structures with direct or indirect influence on individual-level food choices.^19^ In other words, multiple individual and contextual factors interact in several structural subsystems comprising the local environment (neighborhood, city, or town), the food system, the food environment, transport, housing, social protection, and employment systems. From a systems science perspective, the characteristics of the patterns of behavior occurring within the limits of, and the rules (including policies) that govern, these subsystems affect the availability, accessibility, acceptability, and nutritional quality of the food supply.^20^^,^^21^

To illustrate the complexity of factors influencing the social distribution of dietary behaviors (ie, excessive salt consumption), we consider the population groups in a low SEP. These groups are characterized by precarious employment conditions (long or inflexible working hours and insufficient living wages), which are associated with less frequency of meals prepared or eaten at home compared with those in a high SEP.^19^ This can then lead to habitual purchasing of commercial ultra-processed foods high in fat, sugar, and salt or meals supplied at food service settings. In the most marginalized neighborhood areas, the food retail infrastructure and the presence of community networks available to provide meal-based support can also play a key role in the exposure to unhealthy or healthy food and the development of food preferences.

### “Upstream” vs “downstream” public health interventions

Building from the public health research field, a distinction exists between upstream and downstream health-promotion interventions. Interventions centered in “upstream” determinants of health seek to modify aspects of the local environment where people make choices and where population-level patterns can be observed, taking a whole-of-population approach. These include food reformulation, fiscal policies, certain school programs, and massive communication campaigns. Interventions centered in “downstream” determinants target individual behaviors influenced by knowledge, attitudes, and beliefs. The latter may include a strong educational component, are usually small in scale, and are directed to subgroups of the general population.^22^

Some evidence suggests that “upstream” interventions, situated at the macro-level, encompassing regions, nations, and cities, could better reduce health inequities, while “downstream” interventions could potentially increase inequalities.^23^^,^^24^ Downstream-level interventions require high levels of agency on the part of the population and the effects of the intervention are frequently not sustained over time.^23^ In contrast, upstream-level interventions are usually resource-intensive at all stages of program design, including evaluation and monitoring. A recent umbrella review on policies to reduce socioeconomic diet inequalities identified insufficient evidence with reference to salt-reduction initiatives.^25^ Furthermore, a systematic review concluded that multicomponent strategies involving both upstream and downstream interventions achieved the biggest reductions in salt consumption across an entire population.^26^

Since the establishment of the WHO Global Action Plan for the Prevention and Control of NCDs, 154 member states have committed to implementing policy actions to reduce sodium intake, including 9 countries with comprehensive and mandatory policy plans (5% of WHO 194 member states).^7^ Therefore, a new assessment of salt-reduction initiatives could help inform nutrition stakeholders in the use of combined upstream and downstream strategies in the future for the benefit of the whole population. In addition, the evidence gathered from a systematic review could deliver a refreshed perspective to identify the best implementation practices to reduce salt and sodium intake in vulnerable communities. The objective of the current study was to systematically review the literature and assess the impact of salt-reduction initiatives on health and behavioral outcomes of adults by SEP.

## METHODS

Details of the protocol for this systematic review were registered on the International Prospective Register for Systematic Reviews (PROSPERO) as CRD42020183289. A preliminary search of studies was performed to refine the search terms and to identify the least number of databases that would generate as many eligible studies as possible. The key terms consisted of the following, organized in 3 search fields: (1) salt, sodium chloride; (2) socioeconomic, social class; and (3) health promotion, nutrition policies, initiative, campaign, intervention.

Truncation (*) was used where applicable. Key search terms were combined using the Boolean logic “OR” operator; database search fields were combined using the Boolean logic “AND” operator. The search results were expanded by considering the entire body of the document (All text). Filters were applied (ie, Language: English; age: 18–64 years), considering the main concepts examined in the review following the PICCOS structure: Population, Intervention, Comparator, Context, Outcome, and Study design (Table 1).

**Table 1.** PICCOS Criteria for Inclusion of Studies

**Parameter**	Inclusion criteria	Exclusion criteria
Population	Adults aged 25-64 years of age	Adult population subcategories (eg, pregnant women)
Intervention	Salt-reduction interventions, programs, policies	Studies without measurement of dietary or behavioral salt or sodium intake stratified by socioeconomic position (SEP)
Comparator	Before implementation or at baseline measurement and after implementation measurement	Missing measurement outcomes on the impact of the salt-reduction intervention
Context	Studies from lower-, middle- and high-income countries	Studies not published in the English language
Outcome	Change in salt intake, change in behavior related to salt intake or salt use, and change in knowledge, attitudes, or beliefs related to salt intake or salt use	Studies were excluded if missing SEP data and/or salt-intake data
Study design	Systematic reviews, randomized controlled trials (RCTs), cohort studies, case-control studies, cross-sectional studies, longitudinal studies, and case-report studies	Editorials, opinion pieces, study protocols, conference abstracts

Six databases available through subscription at University College Cork from February 16 to 19, 2021, were searched: CINAHL Plus with Full Text, Scopus, Embase, MEDLINE (Ovid), PubMed, and Web of Science Core Collection. As per the protocol, the search was re-run on April 14 and 29, 2022, to identify studies published from February 2021 to April 2022.

### Study selection and inclusion criteria

The initial title and/or abstract screening was conducted independently by 2 reviewers (K.G. and S.G.), based on the inclusion and exclusion criteria specified in Table 1. Following a snowballing approach, reference lists of relevant papers were examined to identify additional studies. Records were imported to Rayyan (Ouzzani et al, Cambridge, MA, USA), which is a web-based tool for screening research articles in collaborative and blinded systematic reviews.^27^ At this stage, queries on issues or conflicts relating to inclusion and exclusion criteria were discussed and resolved between 3 or 4 team members (K.G., S.G., and A.C.N.).

### Data extraction and critical appraisal

Two reviewers collected data independently using a predesigned electronic form. Bibliographic data, abstract, URL, objective(s), study design, study duration, recruitment strategy, participants’ characteristics, study setting, country, type of intervention, outcomes measured, assessment method, and the key conclusions of authors were extracted from each of the included studies in this review.

The studies included were divided by study type for critical appraisal, using the AXIS and RoB 2 tool. The AXIS tool was used to assess the risk of bias in cross-sectional and observational studies and the RoB 2 tool was used to assess the quality of studies with an experimental component (randomized controlled trials [RCTs]). Three reviewers (S.G., K.G., and A.C.N.) independently performed the risk of bias assessment for each of the studies. Aspects from these tools that raised concerns among the reviewers included selection bias in studies focused only on participants with CVD, low attrition or response rate confounding, and/or not including a description of the follow-up strategy and methods used to assess dietary intake. The results from the critical appraisal were discussed by the 3 reviewers (K.G., S.G., and A.C.N.) and conflicts were resolved by consensus. Studies were classified as having low risk of bias, high risk of bias, or some concerns.

### Data analysis

A summary of the study characteristics was presented in a tabulated form. The results were interpreted according to study design, to facilitate comparison between study outcomes, using a narrative synthesis approach.

## RESULTS

**Figure 1.**
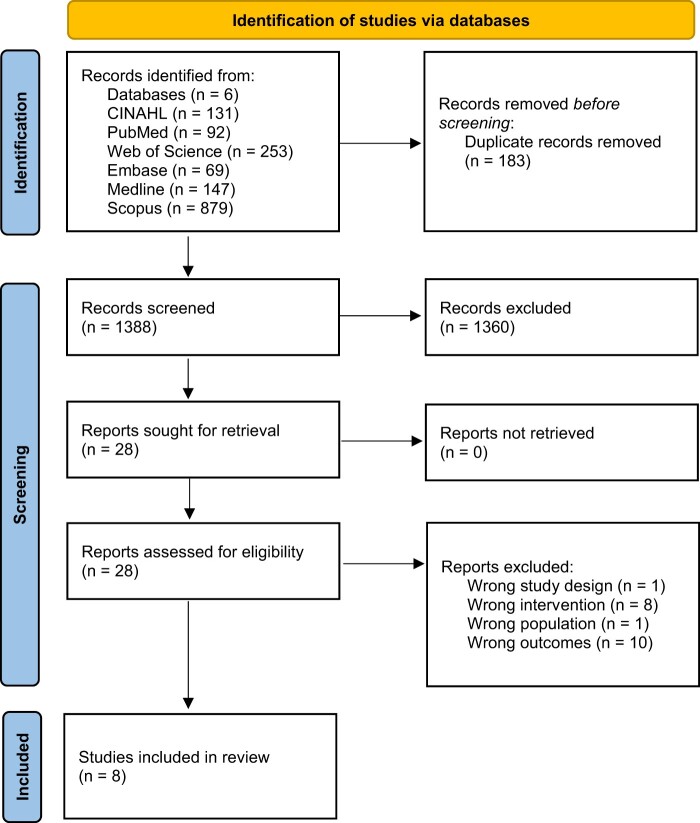
PRISMA (Preferred Reporting Items for Systematic reviews and Meta-Analyses) Flow Diagram

In the search of the literature in 6 library databases, after filters were applied, a total of 1571 records were retrieved (Figure 1). From these records, 1388 studies were screened by reviewing the title and abstract or the full text. Six studies met the inclusion criteria. Two additional studies were identified by performing the search strategy a second time during April 2021. The leading reasons for exclusion were duplicates, incorrect study type, incorrect population, no intervention, wrong outcome, and/or outcome not stratified by SEP. A list of studies that appeared to meet the inclusion criteria but that were excluded can be found in the Table S1.

Overall, this systematic review identified 8 research studies (Table 2).^28–35^ The publication year ranged from 2004 to 2022. The sample size ranged from 117 to 67 980 participants. Five studies were observational, 2 studies had a quasi-experimental design, and 1 study was an RCT. Five studies relied on household data collection,^28–30^^,^^34^^,^^35^ and in 3 studies data were collected in community centers or healthcare settings.^31–33^

**Table 2.** Summary of Included Studies by Study Design

Study, year, country	Sample characteristics	SEP measure	Salt-intake measure	Intervention	Salt-intake outcomes in relation to SEP measure
Observational studies (*n* = 5)					
Ji and Cappuccio, 2014,^28^ United Kingdom	1027 men and women ages 19-64 y (representative of national population)	Educational attainment and occupation	2000-2001: 7-day food diary records and 24 -h urine collection 2008-2011: a 3- or 4-day food diary	Comprehensive salt-reduction program introduced in 2003/2004, based on a mass media awareness campaign; establishment of targets for the content of salt in food products led by the Food Standards Authority; a voluntary industry agreement to reformulate food; and a rolling program to monitor salt (intake/content)	In 2008-2011, the lowest-SEP group had a significantly higher dietary sodium intake than the highest-SEP group. Between the surveys, there was an average reduction in dietary sodium intake of 366 mg/day (0.9 g of salt/day) and the socioeconomic gradient remained unchanged.
Sutherland et al, 2013,^29^ United Kingdom	67 980 individuals using representative survey data	Ethnic group and total annual income	Self-reported salt use at the table	UK salt reduction strategy. In 2003, the UK Food Standards Agency and the Department of Health in England committed to reducing salt intakes to no more than 6 g/day via 2 general strategies: by a gradual reduction in salt content of foods (via the food industry reformulating processed foods) and by increasing consumer awareness of the impact of salt on health.	Compared with adults in the highest-SEP group, adults in the low-SEP group were consistently more likely to add salt at the table across all years. Compared with other ethnic groups, White populations were more likely to add salt at the table across all survey years.
McLaren et al, 2014,^30^ Canada	14 989 Canadian adults >16 y of age, using representative survey data	Income and education	1970-1972 and 2004: sodium intake from 24-h dietary recall and self-reported use of table salt	Since 1982, Canada’s Food Guide encourages to select and prepare foods with limited amounts of fat, sugar, and salt.	No association between income or education and sodium intake in men. In 2004, women in the highest education category consumed more sodium than women in the lowest education category (*P* < .10). The effect did not hold in fully adjusted models and the concentration index was very small.
Donfrancesco et al, 2021,^31^ Italy	3835 men and women aged 35–79 y; residents in all Italian regions	Educational level	24-h urine collection	In 2008, the Italian Ministry of Health, within the framework of the “Gaining Health: Making Healthy Choices Easy” strategic program for NCD prevention, launched specific initiatives, with the collaboration of the Interdisciplinary Working Group for Salt Reduction in Italy, aimed at the reduction of excess salt intake.	In both periods and genders, 24-h sodium excretion was inversely associated with educational levels. The mean sodium excretion in participants with higher education was 181 mmol/day and 159 mmol/day and in those with the lowest education was 191 mmol/day and 175 mmol/day, respectively, in 2008-2012 and 2018-2019.
Strauss-Kruger et al, 2022,^32^ South Africa	668 men and women aged 20-30 y	Ethnicity and socioeconomic status	24-h urinary sodium excretion	South Africa implemented mandatory sodium reduction legislation for selected processed-food categories, first enforced on June 30, 2016.	A reduction of 1.9 g of salt per day was observed in the intake of lowest socioeconomic groups.
Quasi-experimental studies (*n* = 2)					
Archuleta et al, 2012,^33^ United States	117 men and women living in New Mexico	Ethnicity and income	3-day food record	Statewide health-promotion program known as Kitchen Creations: (KC), consisting of four 3-h-long classes spent preparing a meal that the participants ate together	Change in sodium intake was significantly associated with income (*P* = .03). Sodium intake was reduced in the highest-income group (*P* = .006) but not in the middle- or lowest-income groups.
Shankar et al, 2013,^34^ United Kingdom	22 264 individual participants	Income	Spot urinary sodium	The UK salt-reduction campaign, with a 2-pronged strategy. The first, an awareness campaign; the second part of the strategy was product reformulation by working with the food industry.	Participants in the lowest income category had an approximately 39% higher salt intake than those in the highest-income group. The salt campaign was reported to have reduced salt intake by approximately 10%.
Randomized controlled trial (*n* = 1)					
Kaur et al, 2020,^35^ India	668 adults in the family who usually cook food (35-70 y)	Type of housing	Sodium intake change from food-frequency questionnaire data	The intervention group received information technology (IT)–enabled nutrition education, which had 2 components: (1) interpersonal, which included a “SMART Eating” kit (kitchen calendar, dining table mat, and measuring spoons); (2) IT—SMS, e-mail, social networking app, and “SMART Eating” website, over a 6-mo period.	A significant net reduction was observed in salt intake among all 3 SEP groups: –7.5% (*P* = .002) in LIG, –6% (*P* = .01) in MIG, and –1% (*P* = .7) in HIG.

*Abbreviations:* HIG, high-income group; LIG, low-income group; MIG, middle-income group; NCD, noncommunicable disease; SEP, socioeconomic position; SMS, Short Message Service.

The following subsections present the descriptive results from each of the included studies. Table S3 summarizes the effects of these interventions.

### Impact of salt-reduction initiatives in observational studies

Five studies specifically assessed the effectiveness of a national dietary and/or salt-reduction intervention (Table 3).^28–32^ Two different studies conducted by Ji and Cappuccio^28^ and Sutherland et al^29^ in the United Kingdom evaluated the impact of the national salt-reduction program initiated in 2003–2004 by the UK Food Standards Agency and the Department of Health in England. In Canada, McLaren et al^30^ examined sodium consumption before and after the introduction of a statement to the Canadian Food Guide in 1982 “to limit the amount of salt when preparing food.” Across Italy, Donfrancesco et al^31^ assessed the trends on salt intake after the implementation of 2 national salt-reduction strategies. In this study, the Italian national initiatives evaluated were the program for NCD prevention “Gaining Health” co-developed in 2008 by the Italian Ministry of Health and an Interdisciplinary Working Group for Salt Reduction and the National Preventive Plan (NPP) 2014–2019. Finally, Strauss-Kruger et al^32^ conducted research to assess the effectiveness of a mandatory sodium-reduction legislation in South Africa, first enforced on June 30, 2016, with further reductions from June 30, 2019.

**Table 3.** Information on Salt-Reduction Initiatives Implemented at the National Level Identified From Studies Identified in the Systematic Review

Reference No.	Country	Policy/program	Implementation date
28	UK	UK salt-reduction strategy	2003-2004
29	UK	UK salt-reduction strategy	2003
30	Canada	Canadian Food Guide	1982
31	Italy	“Gaining Health” strategic program for NCD prevention and National Preventive Plan (NPP) 2014-2018, extended to 2019	2008 and 2014
32	South Africa	Regulation 214 (R214) of the Foodstuffs, Cosmetics, and Disinfectants Act No. 54 of 1972 in 2013	2016

Evidence from the United Kingdom suggests a slight decrease (–0.9 g) in salt intake per day between 2000–2001 and 2008–2011.^28^ In 2008–2011, the median dietary sodium intake was 2245 mg/day (interquartile range = 1092 mg/day) or 5.6 g of salt/day. Between the surveys, 10 years apart, the lowest SEP group had a significantly higher dietary sodium intake than the highest SEP group. The authors of this study concluded that inequalities in dietary salt intake did not decrease; on the contrary, the gap between more and less affluent groups became greater by 2008–2011 (from a 3.5% difference in dietary sodium intake to a 5.7% difference). Similarly, the results from Sutherland et al^29^ demonstrated that adults in the lowest SEP were consistently more likely to add salt at the table across the years 1997, 1998, 2003, 2005, 2006, and 2007 compared with those in a high or medium SEP. Despite the overall reduction in dietary salt use, both UK studies found that salt intake was most common in adults living in the north and least common in adults living in the south of England.^28^^,^^29^ Additional studies reported this inverse association between SEP and salt intake.^31^^,^^34^ Taking into account the well-established north–south divide in England, where, typically, people from the south are of higher SEP, it is possible that this phenomenon contributes to the higher salt intakes found among adults living in the north.^36^^,^^37^

In Italy, 24-hour sodium excretion was inversely associated with educational levels before and after the implementation salt-reduction initiatives undertaken by the Italian Ministry of Health.^31^ Although the socioeconomic gradient in salt intake remained unchanged, the study reported a reduction in salt intake in Italian men and women of 1.3 g and 1.1 g, respectively (from 10.8 g in men and 8.3 g in women in 2008–2012 to 9.5 g in men and 7.2 g in women in 2018–2019). However, in Canada, McLaren et al^30^ observed the opposite trend in 2004, where women of a higher educational level consumed significantly more sodium than women of a lower educational level.

From the evidence reviewed, the study conducted by Strauss-Kruger et al^32^ reported a moderate decrease of 1.9 g of salt intake per day in lower-income groups in South Africa, suggesting a greater health benefit in this group of the general population following a comprehensive and inclusive salt-reduction initiative. In South Africa, since 2016, it is mandatory to reformulate and reduce sodium in a range of selected processed-food categories. Importantly, in the publication by WHO Europe, entitled “Accelerating Salt Reduction in Europe,” South Africa is considered one of the best examples in adopting a regulatory approach, outside of Europe.^1^ This initiative appears to have achieved a significant reduction of 1.2 g of salt per day from 2013 to 2018, after a 4.56-year follow-up in 311 adults (median salt intake: 7.26 g/day in 2018).

### Impact of salt-reduction initiatives in quasi-experimental studies

Through the systematic review of the literature, 2 different studies reporting salt intake by SEP after an intervention were identified.^33^^,^^34^ Archuleta et al^33^ performed a postintervention evaluation of a health-promotion program consisting of four 3-hour-long cooking classes for adults with type 2 diabetes in New Mexico. After the intervention, participants received questionnaires through the postal service containing a food record survey and voluntarily returned the form. The results showed a decreased sodium intake (from 2882 to 2695 mg/day) in 117 men and women. In addition, in this study conducted among a New Mexican population, sodium intake change was significantly associated with income, in which the highest income group showed the greatest reduction in sodium intake.

Shankar et al^34^ used secondary data to compute change in salt intake after the implementation of the UK national salt-reduction initiative. This latter study reported that participants in the lowest income category had an approximately 39% higher salt intake than those in the highest income group.

### Impact of salt-reduction initiatives in RCTs

We identified 1 RCT conducted in adult populations in India.^35^ The trial included a core nutrition education program to reduce the use and intake of salt. The intervention consisted of providing dietary guidance through a “SMART Eating” website, digital messages (text messages, e-mail, and WhatsApp), and educational materials (eg, kitchen calendar and measuring spoons). After the intervention, the research authors observed a significant decrease in salt intake from baseline—evaluated by using a validated food-frequency questionnaire—in low-income groups (–7.5%), in middle income groups (–6%), and in high-income groups (–1%). In the intervention group (*n* = 366 adults), the overall salt intake postintervention was 7.43 g (SD: 0.13). The results from this health-promotion trial showed a substantial decrease in salt intake in the low-income group.

### Critical appraisal

Table S2 shows the results from the critical appraisal. Studies had some concerns related to missing numerical data shown in figures in the Results section^28^ and providing a clear and detailed description of the methodology, particularly with reference to the authors’ decisions related to the treatment and interpretation of nonresponders^29^^,^^30^ and to the target (reference) population.^33^

## DISCUSSION

From this small sample of research studies (*n* = 8) reporting changes in salt or sodium intake by SEP after the implementation of a salt-reduction intervention, 6 studies observed overall reductions in salt or sodium intake. Recent systematic reviews centered in evaluating the effectiveness of salt-reduction policies on behavioral and dietary outcomes identified as many as 70 empirical and modeling studies and 96 different national salt-reduction strategies.^26^^,^^38^^,^^39^ However, the present systematic review indicates a critical gap in the literature given the need to assess the impact of salt-reduction initiatives on health and diet by SEP indicators to address health inequalities.

In line with the WHO Europe information, the evidence from the United Kingdom and Italy confirms that population groups in the lowest SEP tend to have a higher salt intake compared with those in the highest SEP.^1^^,^^28–31^^,^^23^^,^^26^ In particular, the United Kingdom is an example of a successful salt-reduction strategy targeting the “upstream” determinants of salt consumption. The engagement of the food industry in the reformulation of products, promoted and monitored by the Food Standards Agency, achieved reductions of 70% in a range of commercially available foods and contributed to an average reduction of 1.4 g of salt per day in 7 years.^1^

Furthermore, important to the aim of this systematic review, there were 2 research studies conducted in South Africa and India each indicating a greater reduction in salt use or intake postintervention in the low-SEP groups than in the high-SEP groups.^32^^,^^35^ These studies differ in their salt-reduction program implementation approach, as the study in South Africa is based on a mandatory legislation to reduce the content of salt in processed foods and the study in India refers to a health-promotion trial for improvement in dietary habits. What we can learn from these 2 studies is that using evidence-based information for public action is conducive to benefits, however small, in population health.^26^

From the initiatives in South Africa and the United Kingdom, we identified a potential to effectively reduce inequalities in dietary salt intake across SEP groups. In these countries, the salt-reduction initiatives are based on a whole-of-society approach, and therefore, a multicomponent and upstream program, that engages the food industry with public health plans (eg, in the reformulation of processed food). From the South African regulatory policy adoption experience, the evidence indicates that changing the nutritional quality of widely consumed products available in the commercial food environment can lead to a modest reduction in salt intake (1.2 g/day) in less than 5 years from commencement of law enforcement. Processed foods (such as bread, cured meats, and cheese) contribute up to 80% of total dietary salt intake, emphasizing the importance of adopting regulatory approaches, where feasible, and integrating action from food industry stakeholders into NCD prevention initiatives.^38^^,^^40^

Yet, none of the research studies assessed reported a large salt reduction, such as that reported in Finland or Japan (“about 3 g of salt per day reduction achieved among the population over two decades”).^1^ To achieve this, the Finnish government applied multiple upstream strategies, including nutrition labeling legislation, reformulation of products by replacing sodium chloride with nonsodium alternatives, public nutrition education campaigns, and the establishment of epidemiologic surveillance of dietary salt intake.^38^ However, to our knowledge, the published literature reporting effects of salt-reduction initiatives in these 2 exemplary countries has not been analyzed by SEP.

### Limitations

The systematic review has a clear limitation, since the search of articles included those published in the English language, constraining the wealth of the evidence identified. It is important to note that many relevant articles were excluded for not reporting outcome measures by SEP, although these studies included baseline data reported by SEP. Furthermore, many articles did not aim to measure outcomes of a salt-reduction initiative, even in those subgroups of the population who are at risk of experiencing nutritional and/or health inequities. In the health inequities field, it is important to engage with vulnerable groups in the administration and completion of population health surveys. If there is a low response rate, the living condition of the vulnerable population can be underrepresented in the overall data. An additional limitation is that the study designs and the outcomes reported in the included research articles differ, which limits the statistical comparability between outcome measures.

## CONCLUSION

To our knowledge, this is the first systematic review performed with the aim to identify all research that has been published on the topic of salt-reduction initiatives and socioeconomic health inequalities among adults. The discourse focused on addressing the existing structural determinants of health inequities was well established after 2008, following the WHO report from the Commission on Social Determinants of Health entitled “Closing the Gap in a Generation.”^41^ Marginalized communities and vulnerable populations can be targeted by improving national and regional food environments, and through the introduction of intersectoral programs addressing all dimensions of nutrition and health, such as those that are focused on upstream determinants and food and health policies.

The research published on the impact of salt-reduction initiatives disaggregated by SEP indicators is very limited. In 2019, as many as 96 WHO member states had an initiative in place; however, this systematic review found that the monitoring efforts of national salt-reduction programs, strategies, and initiatives are not conducted to identify if there are adult population groups who are not being targeted effectively. This is of concern when evidence suggests that lower-income populations have a higher consumption of salt/sodium than the rest of the population. The articles included in the systematic review also showed that, when the research outcomes are stratified by SEP, there is a higher effect in lower SEP groups, suggesting potentially greater health benefits to be gained in a group that is usually at higher risk of CVD.

## Supplementary Material

nuae088_Supplementary_Data
