# Supplementary material for: Systematic Review on the Impact of Salt-Reduction Initiatives by Socioeconomic Position to Address Health Inequalities in Adult Populations
Source: Nutr Rev. 2024 Jul 8;83(3):e1090–100. doi: 10.1093/nutrit/nuae088 (PMC11819476; doi:10.1093/nutrit/nuae088)
Supplement: nuae088_Supplementary_Data [file nuae088_supplementary_data.zip › nuae088_Supplementary_Data/jnutr-SLR title page.docx]

**Title Page**

**Title**:

Systematic review on the impact of salt reduction initiatives by socioeconomic position to address health inequalities in adult populations

**Author Names**:

Ana Contreras Navarro, DSc, BSc

Kerrie Gallagher, BSc, BA

Sally Griffin, BSc

Clarissa L. Leydon, MSc, BSc

Ivan J Perry, PhD, MSc, MD

Janas M. Harrington, PhD, MA, BSc

**Author Affiliations**:

A. Contreras Navarro, K. Gallagher, I.J. Perry, C.L. Leydon and J.M. Harrington are with the School of Public Health, University College Cork, Cork, Ireland. S. Griffin was with the School of Public Health, University College Cork, Cork, Ireland and now is with the Leeds Beckett University, Leeds, West Yorkshire, England.

**Corresponding Author**:

Ana Contreras Navarro, 4th Floor, Western Gateway Building, Western Road, Cork, Ireland, T12XF62, +353(0)214205500, acontrerasnavarro@ucc.ie
