# Supplementary material for: Systematic Review on the Impact of Salt-Reduction Initiatives by Socioeconomic Position to Address Health Inequalities in Adult Populations
Source: Nutr Rev. 2024 Jul 8;83(3):e1090–100. doi: 10.1093/nutrit/nuae088 (PMC11819476; doi:10.1093/nutrit/nuae088)
Supplement: nuae088_Supplementary_Data [file nuae088_supplementary_data.zip › nuae088_Supplementary_Data/jnutr-SLR_supporting_information.docx]

# Supporting Information

**Table S1.** List of studies that appeared to meet the inclusion criteria, but which were excluded.

| **Reference** | **Reason for exclusion** |
| --- | --- |
| Azadnajafabad S, et al. Disparities and spatial variations of high salt intake in Iran: a subnational study of districts based on the small area estimation method. Public Health Nutr. 2021 | Not an intervention study |
| Charlton K, et al. Leveraging ongoing research to evaluate the health impacts of South Africa's salt reduction strategy: a prospective nested cohort within the WHO-SAGE multicountry, longitudinal study. BMJ open. 2016 | Not stratified by SES |
| Chen X, et al. Global burden attributable to high sodium intake from 1990 to 2019. Nutr Metab Cardiov Dis. 2021 | Not an intervention study |
| De Magistris T, et al. Effects of the nutritional labels use on healthy eating habits in Spain. Agricul Econ. 2010 | Not an intervention study |
| Gray L, Leyland AH. A multilevel analysis of diet and socio-economic status in Scotland: investigating the ‘Glasgow effect’. Public Health Nutr | Not an intervention study |
| Ji C, Cappuccio FP. Socioeconomic inequality in salt intake in Britain 10 years after a national salt reduction programme. BMJ open. 2014 | Not stratified by SES |
| Kisioglu AN, Aslan B, Ozturk M, Aykut M, Ilhan I. Improving control of high blood pressure among middle-aged Turkish women of low socio-economic status through public health training. *Croat Med J*. 2004 | Not stratified by SES |
| Layeghiasl M, Malekzadeh J, Shams M, Maleki M. Using social marketing to reduce salt intake in Iran. Front Public Health. 2020 | Effect of intervention not stratified by SES |
| Nichols S, et al. Socio-demographic factors in relation to habitual sodium and potassium intakes among adults in Trinidad and Tobago. Nutrition and Health. 2022 | Not an intervention study |

**Table S2.** Critical appraisal of included studies organised by study design.

Experimental studies using RoB-2 tool (n=1).

| Lead author | Year | Randomization process | Deviations from the intended interventions | Missing outcome data | Measurement of the outcome | Selection of the reported result | Overall |
| --- | --- | --- | --- | --- | --- | --- | --- |
| Kaur | 2020 | 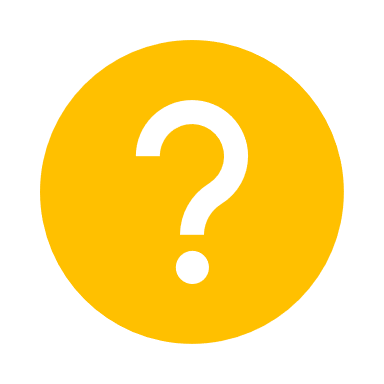 | 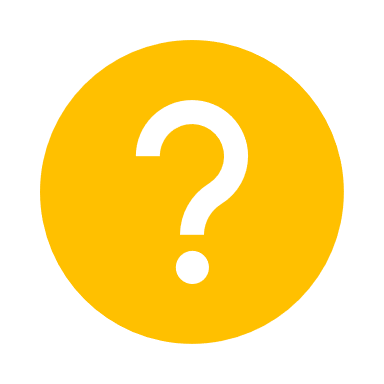 | 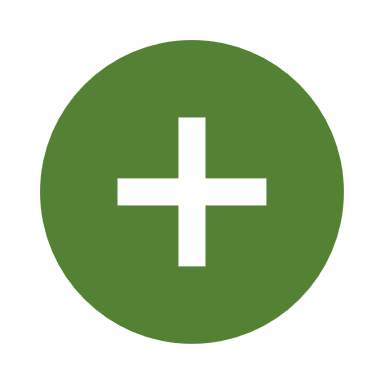 | 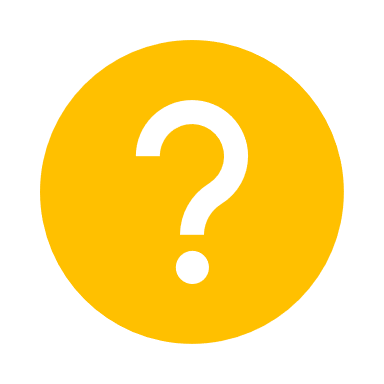 | 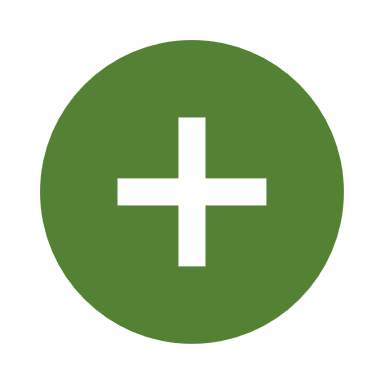 | 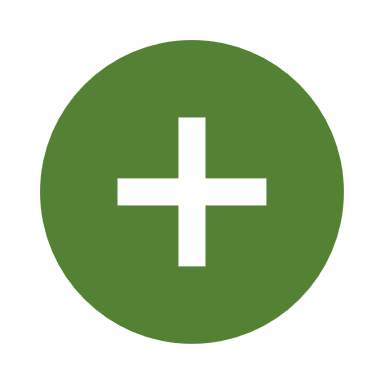 |

Observational studies and quasi-experimental studies using AXIS tool (n=7).

| Lead author | Year | Clear objectives | Appropriate methodology | Results adequately described and internally consistent | Discussion justified by the results | Other (conflicts of interest and consent) | Overall |
| --- | --- | --- | --- | --- | --- | --- | --- |
| Archuleta | 2012 | 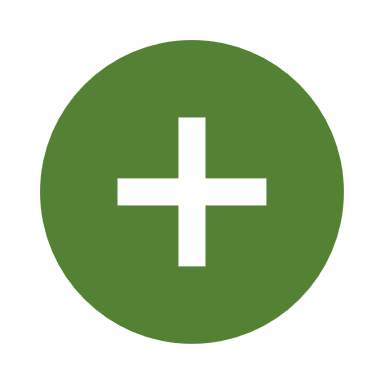 | 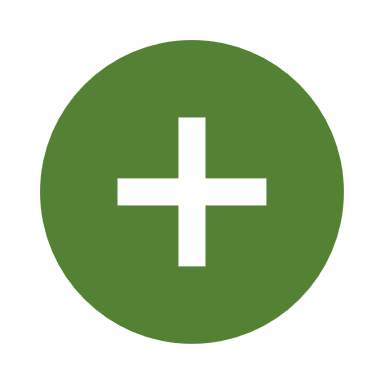 | 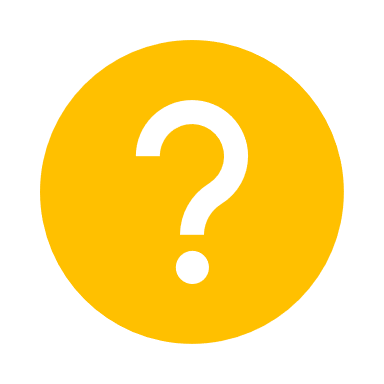 | 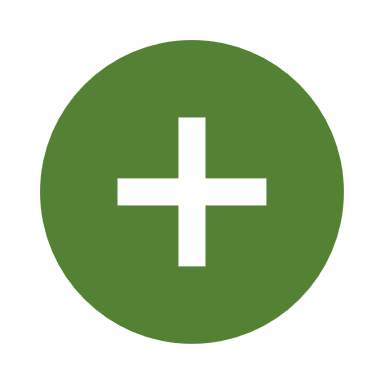 | 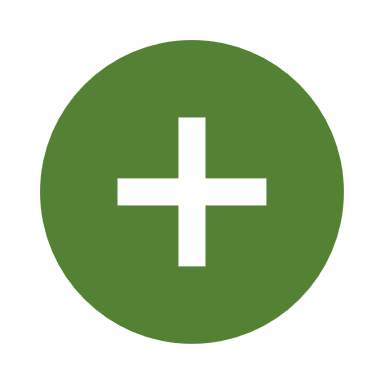 | 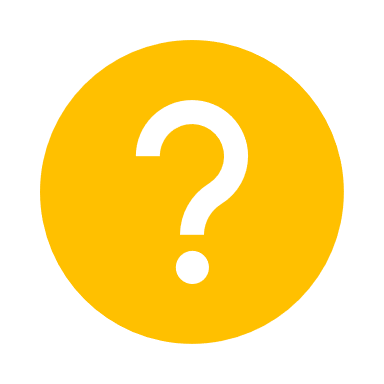 |
| Donfrancesco | 2021 | 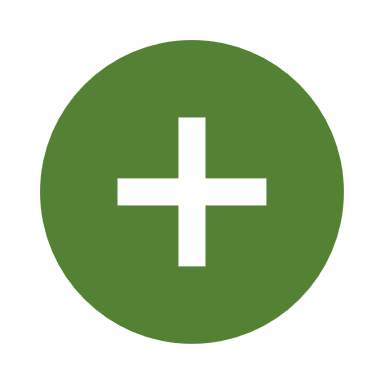 | 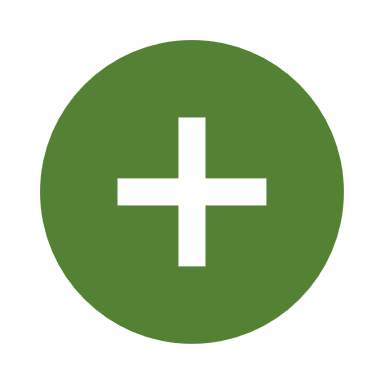 | 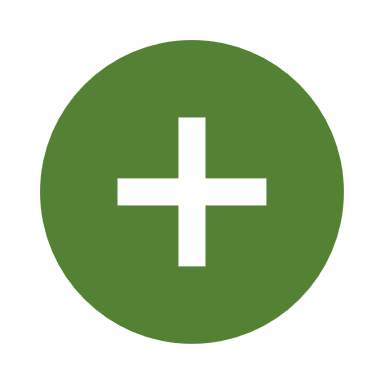 | 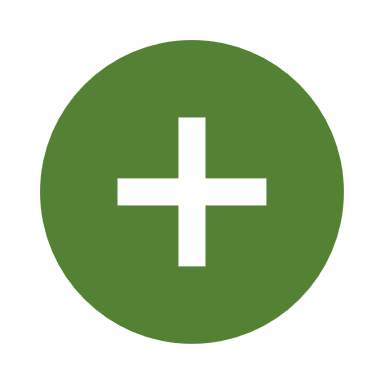 | 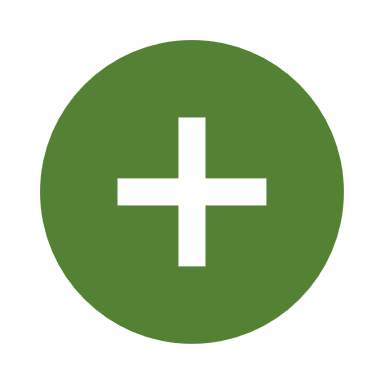 | 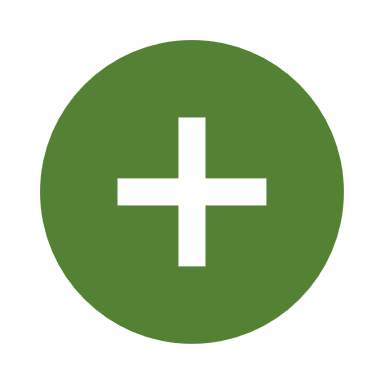 |
| Ji | 2014 | 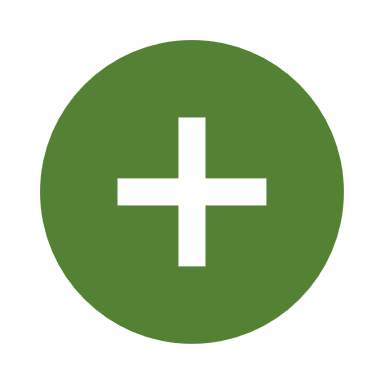 | 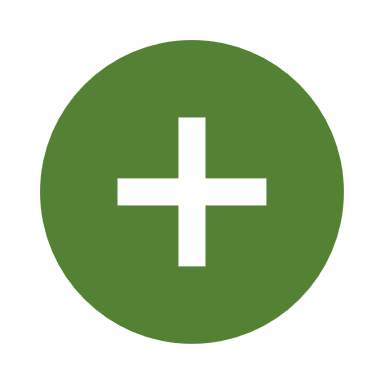 | 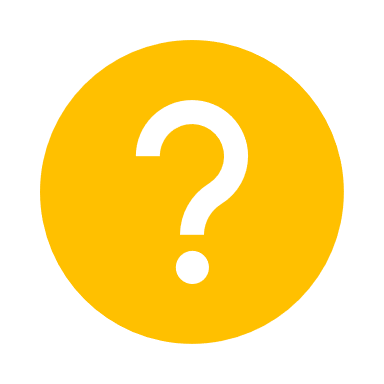 | 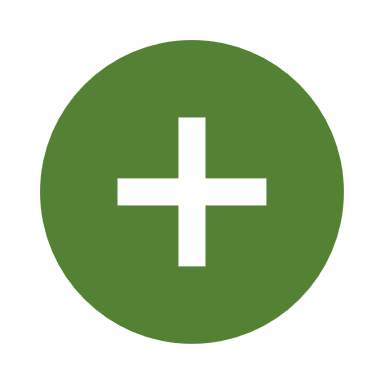 | 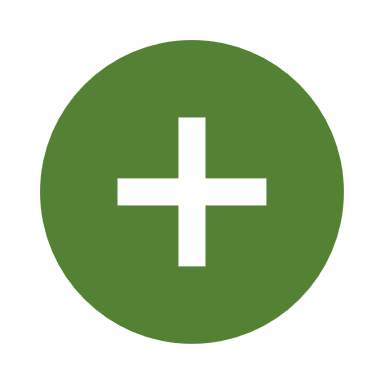 | 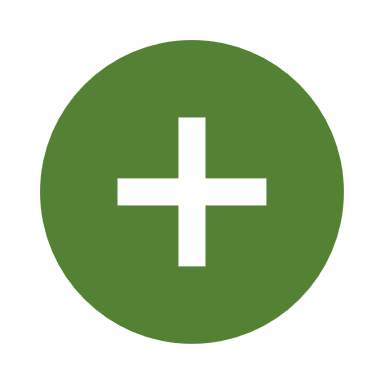 |
| McLaren | 2014 | 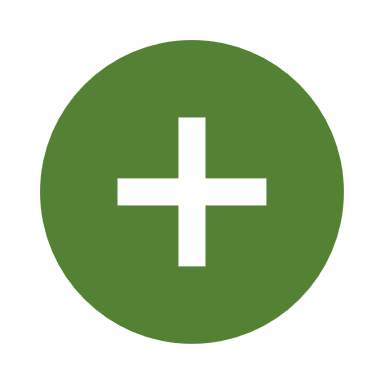 | 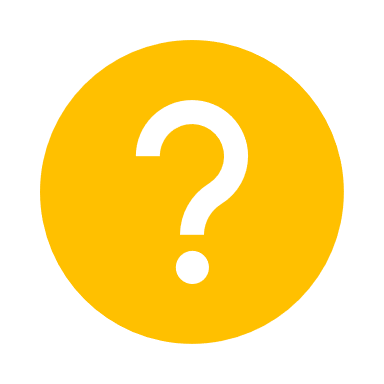 | 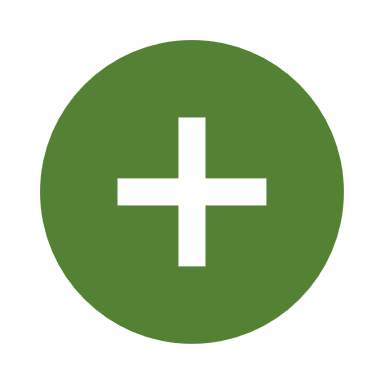 | 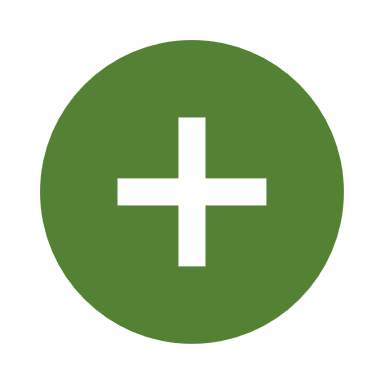 | 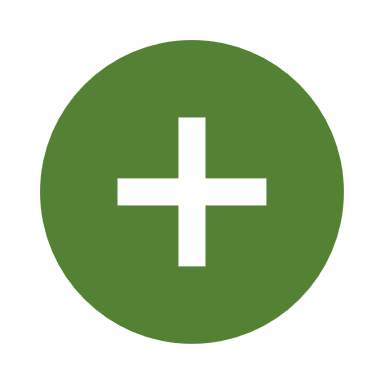 | 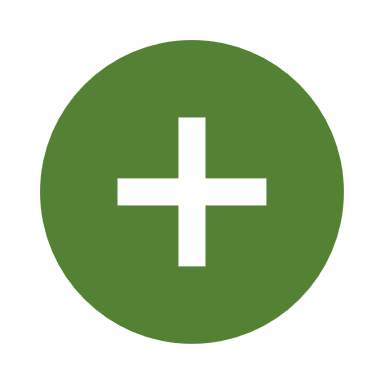 |
| Shankar | 2013 | 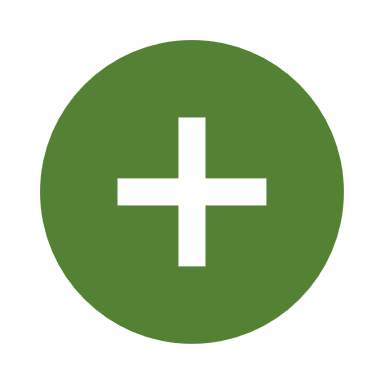 | 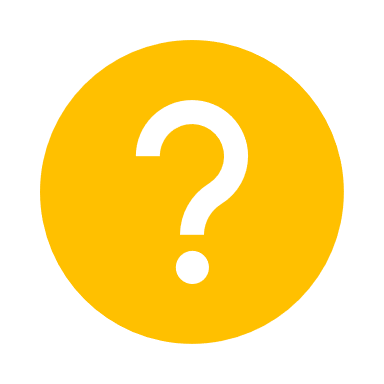 | 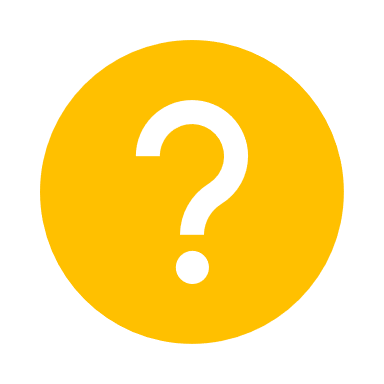 | 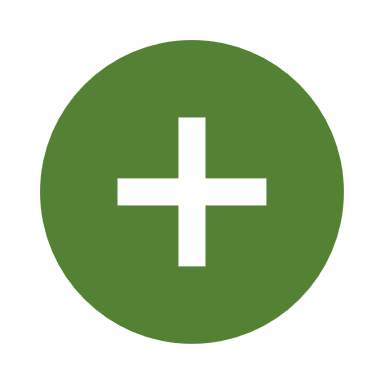 | 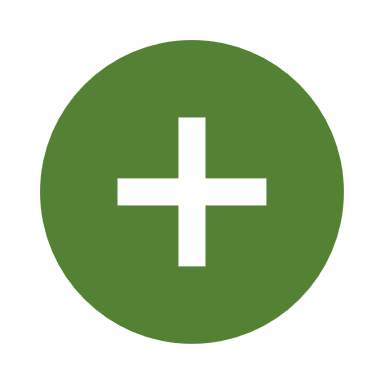 | 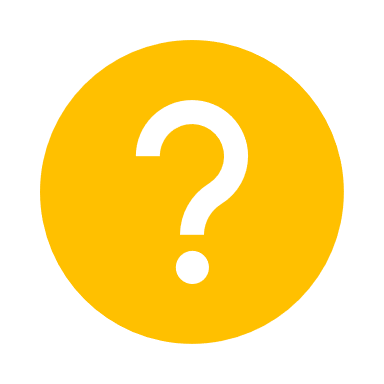 |
| Strauss-Kruger | 2021 | 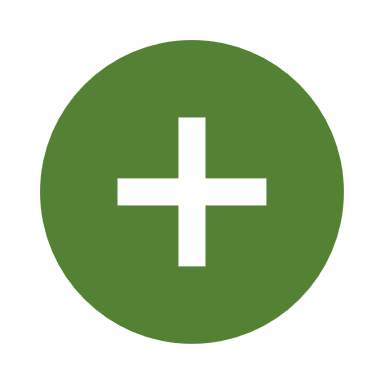 | 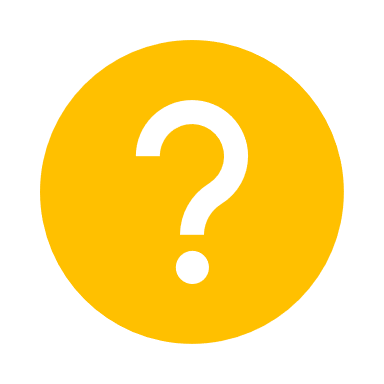 | 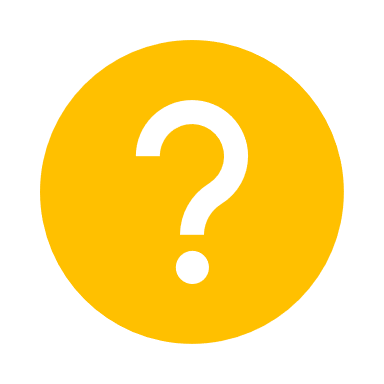 | 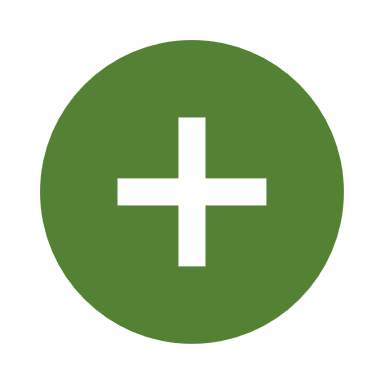 | 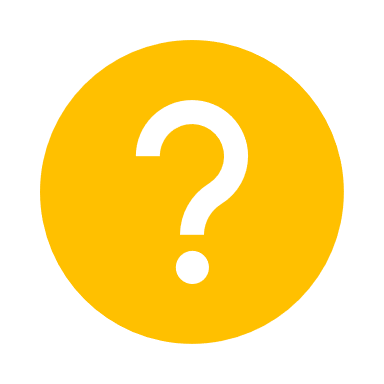 | 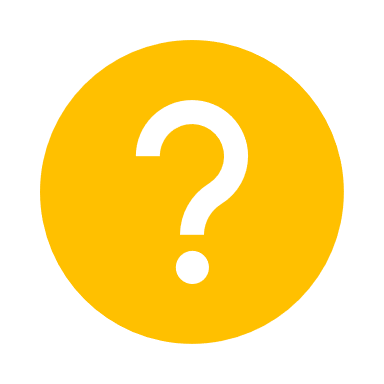 |
| Sutherland | 2013 | 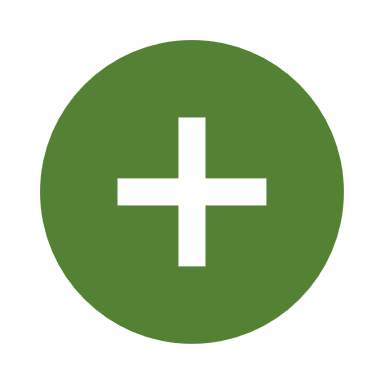 | 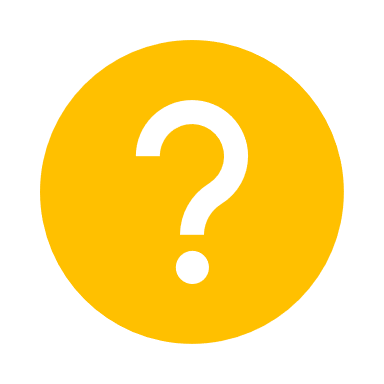 | 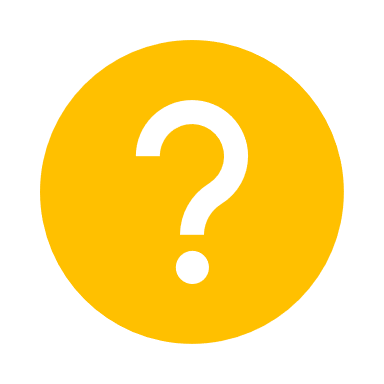 | 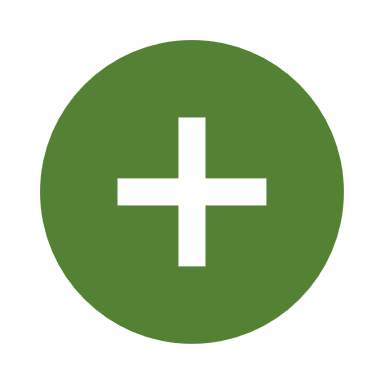 | 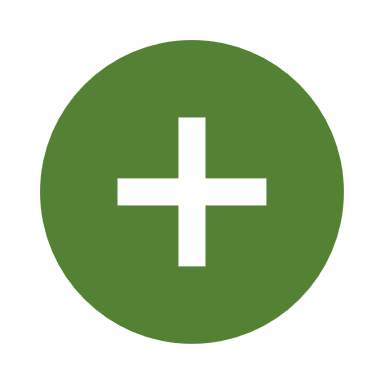 | 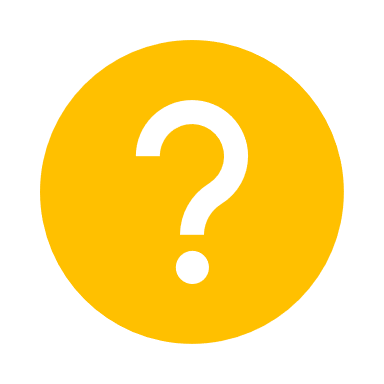 |

Judgement:
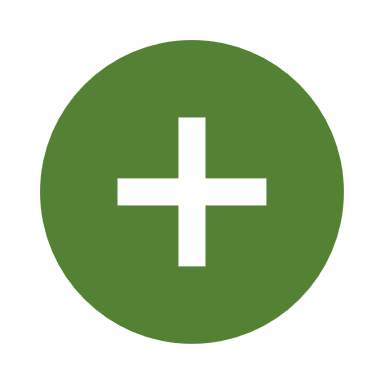
 Low risk of bias,
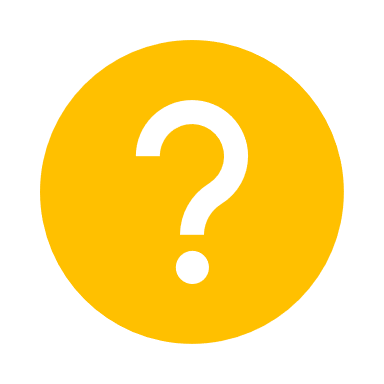
 Some concerns/Not mentioned,
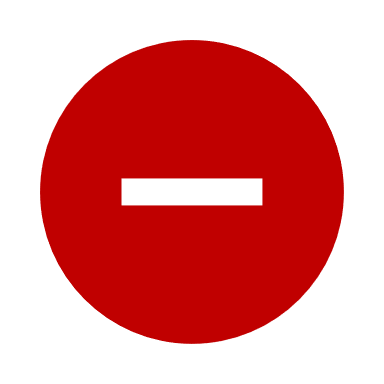
 High risk of bias

**Table S3.** Summary of effects of intervention in each included study (n=8).

| Reference number in main text | Country | Effect | Effect by SEP |
| --- | --- | --- | --- |
| 28 | United Kingdom | **Reduction** in dietary sodium intake of 366 mg/day (0.9 g of salt/day). | Before and after intervention, the **lowest** SEP group had **higher** dietary sodium intake than the highest SEP group. |
| 29 | United Kingdom | **Reduction** in proportion of adults adding salt at the table. | Adults in the **low-SEP** group were **more likely to add salt at the table** between 2003 and 2007. |
| 30 | Canada | NA | There was no evidence of an inequity in sodium consumption (mg/day) in 2004, for men or women. |
| 31 | Italy | **Reduction** in average sodium excretion levels in both high- and low-income levels and genders. | Sodium excretion was **inversely associated** with educational levels. |
| 32 | South Africa | **Reduction** 1.2 g of salt per day in <5 years. | **Lowest** socioeconomic group had a **greater** reduction in salt intake of 1.9 g/d. |
| 33 | United States | NA | Sodium intake reduced in the **highest** income group, but not in the middle- or lowest-income groups. |
| 34 | United Kingdom | **Reduction** in salt intake by approximately 10%. | **Lowest** income group had about 39% higher salt intake than those in the highest income group. |
| 35 | India | **Reduction** in salt intake after 6-months of intervention. | The **lowest**-income group had the greatest reduction of 7.5% in salt intake, compared to 6% and 1% in middle- and high-income groups respectively. |

References

S1. Ji C, Cappuccio FP. Socioeconomic inequality in salt intake in Britain 10 years after a national salt reduction programme. BMJ Open. 2014;4(8):e005683.

S2. Sutherland J, Edwards P, Shankar B, Dangour AD. Fewer adults add salt at the table after initiation of a national salt campaign in the UK: a repeated cross-sectional analysis. Br J Nutr. 2013;110(3):552-8.

S3. McLaren L, Heidinger S, Dutton DJ, et al. A repeated cross-sectional study of socio-economic inequities in dietary sodium consumption among Canadian adults: implications for national sodium reduction strategies. Int J Equity Health. 2014;13(1):1-4.

S4. Donfrancesco C, Noce CL, Russo O, et al. Trend of salt intake measured by 24-h urine collection in the Italian adult population between the 2008 and 2018 CUORE project surveys. Nutr Metab Cardiovasc Dis. 2021;31(3):802-13.

S5. Strauss-Kruger M, Wentzel-Viljoen E, Ware LJ, et al. Early evidence for the effectiveness of South Africa’s legislation on salt restriction in foods: The African-PREDICT study. J Hum Hypertens. 2022:1-8.

S6. Archuleta M, VanLeeuwen D, Halderson K, et al. Cooking schools improve nutrient intake patterns of people with type 2 diabetes. J Nutr Educ Behav. 2012;44(4):319-25.

S7. Shankar B, Brambila‐Macias J, Traill B, et al. An evaluation of the UK Food Standards Agency's salt campaign. Health Econ. 2013;22(2):243-50.

S8. Kaur J, Kaur M, Chakrapani V, et al. Effectiveness of information technology–enabled ‘SMART Eating’ health promotion intervention: A cluster randomized controlled trial. PLoS One. 2020;15(1):e0225892.
